# Viewing medium affects arm motor performance in 3D virtual environments

**DOI:** 10.1186/1743-0003-8-36

**Published:** 2011-06-30

**Authors:** Sandeep K Subramanian, Mindy F Levin

**Affiliations:** 1 School of Physical and Occupational Therapy, McGill University, 3654 Promenade Sir William Osler, Montreal, Qc. H3G 1Y5, Canada; 2 Feil-Oberfeld JRH/CRIR Research Centre, Jewish Rehabilitation Hospital site of Centre for Interdisciplinary Research in Rehabilitation of Greater Montreal, 3205 Place Alton Goldbloom, Laval, H7V 1R2, Canada

## Abstract

**Background:**

2D and 3D virtual reality platforms are used for designing individualized training environments for post-stroke rehabilitation. Virtual environments (VEs) are viewed using media like head mounted displays (HMDs) and large screen projection systems (SPS) which can influence the quality of perception of the environment. We estimated if there were differences in arm pointing kinematics when subjects with and without stroke viewed a 3D VE through two different media: HMD and SPS.

**Methods:**

Two groups of subjects participated (healthy control, n = 10, aged 53.6 ± 17.2 yrs; stroke, n = 20, 66.2 ± 11.3 yrs). Arm motor impairment and spasticity were assessed in the stroke group which was divided into mild (n = 10) and moderate-to-severe (n = 10) sub-groups based on Fugl-Meyer Scores. Subjects pointed (8 times each) to 6 randomly presented targets located at two heights in the ipsilateral, middle and contralateral arm workspaces. Movements were repeated in the same VE viewed using HMD (Kaiser XL50) and SPS. Movement kinematics were recorded using an Optotrak system (Certus, 6 markers, 100 Hz). Upper limb motor performance (precision, velocity, trajectory straightness) and movement pattern (elbow, shoulder ranges and trunk displacement) outcomes were analyzed using repeated measures ANOVAs.

**Results:**

For all groups, there were no differences in endpoint trajectory straightness, shoulder flexion and shoulder horizontal adduction ranges and sagittal trunk displacement between the two media. All subjects, however, made larger errors in the vertical direction using HMD compared to SPS. Healthy subjects also made larger errors in the sagittal direction, slower movements overall and used less range of elbow extension for the lower central target using HMD compared to SPS. The mild and moderate-to-severe sub-groups made larger RMS errors with HMD. The only advantage of using the HMD was that movements were 22% faster in the moderate-to-severe stroke sub-group compared to the SPS.

**Conclusions:**

Despite the similarity in majority of the movement kinematics, differences in movement speed and larger errors were observed for movements using the HMD. Use of the SPS may be a more comfortable and effective option to view VEs for upper limb rehabilitation post-stroke. This has implications for the use of VR applications to enhance upper limb recovery.

## Introduction

Virtual Reality (VR) is increasingly being used as a delivery system for rehabilitation of upper and lower limb impairments and activities of daily living post-stroke [1-3]. VR is a multisensorial experience that allows the user to interact with objects or events in a computer generated virtual environment (VE) [4]. VR provides a platform to design specific individually tailored activities [2] combining factors including intensity, variability, specificity and salience of practice identified as pertinent to enhance experience-dependant neural plasticity [5]. Salient tasks can be practiced in an interesting manner with sustained attention for longer durations in VEs [6].

Virtual reality applications are well-suited to shaping motor output by providing optimal learning conditions that combine extrinsic sensory feedback from the environment with intrinsic sensory feedback from the moving limb [7-9]. Since the quality of the viewing environment may alter how movement is produced [10,11], it is essential to know whether movements performed in a VE are similar to those performed in an equivalent physical environment (PE). Kinematics of pointing, reaching and grasping movements made in 2D and 3D VEs have been compared to those made in PEs in a series of studies by Levin and colleagues [12-15] in healthy subjects and in those with chronic post-stroke hemiparesis. However, the effect of the viewing media on movement kinematics has not previously been addressed.

In a previous pilot study, intensive task-specific practice of pointing movements performed in a 3D VE viewed via HMD for 10 days resulted in an increased range of shoulder flexion and decreased trunk forward displacement, compared to practice in a similarly designed PE in subjects with chronic post-stroke hemiparesis [16]. However, that study could not determine whether post-practice differences in arm and trunk movement patterns were attributable to the training effects or to differences in how the environment was perceived.

In addition to the decrease in the visual quality of objects in a virtual compared to a physical environment, HMDs typically have reduced fields of view (FOV; ~40° horizontal and 30° vertical) [17,18]. Normal adult FOV spans approximately 200° horizontally and 120° vertically, taking both eyes into account [19]. Reducing the FOV to 23° of monocular central vision using pin-holes in a PE led to slower reaching movements and the use of wider grasp apertures in control subjects performing a midline reach-to-grasp (RTG) task [20]. In another study, when viewing a scene through apertures of different sizes cut out of a rectangular box, healthy subjects made larger variable errors with decreasing FOV (4°, 16° binocular view) when pointing towards targets [21]. In addition, endpoint peak velocities were lower and deceleration times for a midline RTG task were prolonged.

It is unclear how distance perception may be affected by viewing a scene through a HMD. For example, reduction in FOV to 47° horizontal × 43° vertical using a simulated HMD did not alter distance perception according to verbal report and when walking towards a remembered target [18]. On the other hand, distance to spheres presented in the midline in a 3D VE were significantly underestimated (35-55%) in healthy subjects wearing an HMD compared to viewing the same scene via a large screen projection system (SPS) [22]. Similarly, distance underestimation (around 30%) was noted in VEs viewed via a HMD for a midline beanbag throwing task compared to real-world throwing [23]. Distance underestimation was also found when control subjects either walked to remembered locations [24] or performed a triangulated walking task (walking along a straight path oblique to the target and then turning to face the target or walk towards it) [25] in a 3D VE viewed using HMD (42° horizontal FOV) compared to the real world.

Part of the distance compression effects occurring with HMDs may be due to mechanical factors like the weight of the helmet [25]. Weight added to the head changes head and neck posture [26] which may cause an increase in the angle formed between eye level and the target leading to distance underestimation [27]. Compared to newer generation HMDs weighing about 5 - 23 oz, older generation HMDs weigh around 28-36 oz and can cause head and neck stress. Other limitations of HMDs for viewing 3D fully immersive environments include: 'cybersickness' (nausea, dizziness, vomiting); visual problems like reduced binocular acuity and eyestrain [28] and a higher incidence of disorientation compared to other display media like a computer monitor, SPS and the reality theatre [29].

The effects of viewing a 2D VE (IREX, Integrated Rehabilitation & Exercise System, GestureTek Inc., Toronto) through a HMD or a computer monitor were compared in young and older subjects by Rand et al [30]. Viewing the VE on a computer monitor resulted in a better sense of presence, faster movements and decreased self-reported perceived exertion. However this study did not evaluate motor performance in terms of movement kinematics. To address the issue of whether perception of the VE viewed through different display media affects how movement is performed (i.e., the quality of movement), a direct comparison between movements made when viewing the environment through HMDs and other viewing media has been suggested [31]. Such a comparison will inform the correct choice of medium for rehabilitation, when promotion of better movement patterns is the goal. Thus, the objective of our study was to estimate if there were differences in movement kinematics when healthy subjects and subjects with chronic stroke made pointing movements to targets in a 3D VE viewed through a HMD or on a SPS. Preliminary results have appeared in abstract form [32].

## Methods

Two groups of subjects participated: individuals with chronic post-stroke (n = 20) hemiparesis and healthy controls (n = 10). Participants with chronic post-stroke hemiparesis were included if they had i) sustained a single unilateral stroke ≥ 6 months previously; ii) had a recovery stage score ≥ 3/7 in the Arm Section of the Chedoke McMaster Stroke Assessment [33]; iii) had no hemispatial neglect, apraxia or major cognitive deficit as assessed by standard clinical tests and iv) could understand simple instructions in English and/or French. Participants were excluded if they had a) sustained a stroke in the brainstem or cerebellar areas; b) any other neurological or orthopedic conditions affecting the arm and/or trunk; and c) claustrophobia, as they would be unable to wear the HMD. The control group consisted of 10 age-matched right-hand dominant subjects recruited from the community with no orthopedic and/or neurological problems.

For the participants with stroke, upper limb impairment was evaluated using the Upper Limb Section of the Fugl-Meyer Assessment (FMA) [34,35] and the Composite Spasticity Index (CSI) [36,37]. The stroke participants were divided into two subgroups according to their upper limb impairment level: mild (FMA score ≥ 50/66) and moderate-to-severe (FMA score ≤ 49/66) [38,39]. All participants signed informed consent forms approved by the Centre for Interdisciplinary Research in Rehabilitation of Greater Montreal (CRIR).

### VR System and viewing media

The VR system consisted of different components connected to a common computer running the CAREN (Computer Assisted Rehabilitation Environment; Motek BV, Amsterdam) VR simulation system. The components were an Optotrak Certus Motion Capture System (Northern Digital Corp., Waterloo, Ont) and an IBM compatible PC (Dual Xeon 3.06 GHz, 2 GB RAM, 160 GB hard drive) running Windows XP. A graphics card (a dual head Nvidia Quatro FX 3000) provided stereoscopic visual representation of the environment with high frame rates (70 Hz). The viewing media used were a HMD (Kaiser XL-50, Kaiser Electro-Optics Inc., Carlsbad, CA) and a SPS. The HMD had a FOV of 50° diagonal, 30° vertical and 40° horizontal, XGA resolution 1024 horizontal pixels × 768 vertical lines, frequency 60 Hz and weighed 1 kg. The HMD blocked all peripheral vision with only the VE visible to the participants. The SPS was 2 m long × 1.5 m high screen with rear projection from 2 projectors viewed using polarized glasses. Peripheral vision of the PE was blocked by a black felt cloth attached to the glasses. This ensured compatibility between the viewing media. Participants wore a baseball cap (220 g) while using the SPS. Head movements were tracked using a 6 marker rigid body attached to the helmet or cap.

### Virtual Environment

**Figure 1:**
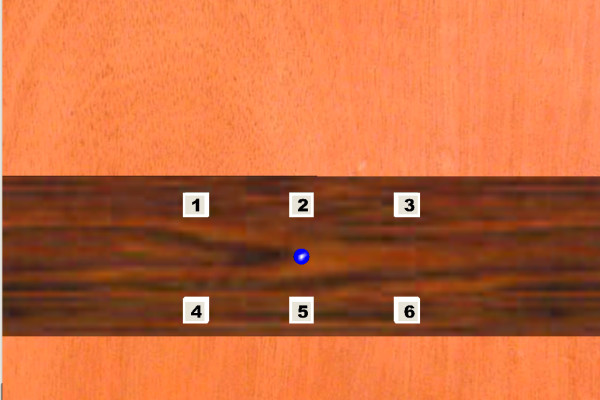
**The virtual environment scene, viewed through the head-mounted display and the large screen projection during arm pointing tasks, consisting of six targets arranged in two rows in the ipsilateral, central and contralateral arm workspace**. The subject's finger position was indicated by the blue dot (circle in centre of targets).

The VE represented an interior elevator scene, consisting of six 36 cm^2 ^(6 cm × 6 cm) targets (numbered 1-6) placed in the midline, ipsilateral and contralateral arm workspaces. The VE was designed to be ecologically valid, with walls and ceiling visible within the environment giving the impression of a closed space. Use of a closed environment was preferred over an environment with open spaces (without walls) by subjects participating in experiments involving VR [40] as it provided better depth cues. Targets 1-3 and 4-6 were positioned in upper and lower rows (Figure 1) separated by a centre-to-centre distance of 26 cms. The distance to the middle target was equal to the subject's arm length, measured from the superolateral border of the shoulder acromion process to the index finger tip. The middle target was aligned with the sternal xiphoid process. The positions and orientations of the head and arm were estimated from the rigid body on the helmet/cap and the endpoint marker (index fingertip respectively). Data were used in real-time to update the scene according to head position.

### Data recording and analysis

Six markers (infrared emitting diodes, IREDs) were placed on anatomical landmarks of the hand, arm and trunk to record kinematic data: index fingertip, dorsomedial border of the wrist crease, lateral epicondyle, ipsilateral and contralateral acromion processes and junction of upper and middle third of the sternum. Data were recorded with an Optotrak Certus system (sampling rate: 100 Hz). Subjects were assigned by block randomization to perform the pointing movement viewing the VE first through either the HMD or the SPS. For both conditions, they were seated on a comfortable chair, with the hips and knees flexed to 90°. The healthy group used their self-reported dominant upper limb and stroke groups used their more-affected upper limb.

In the initial position, the index finger was held at the level of the sternal xiphoid process. The shoulder was in slight abduction (20°) and internal rotation. The elbow was flexed to 90°, the forearm was fully pronated and the wrist was in the neutral position with the fingers semi-flexed. The participants performed 48 pointing movements (8 trials to each of 6 targets, randomized) divided into two blocks of 24 trials each, with rest periods in between. Prior to each trial, an auditory command indicated which target to point at. Subjects were instructed to *"point as quickly and as close to the target as possible"*.

Kinematic outcomes were measured at two levels. At the motor performance level, measures were movement precision [absolute error: root mean square (RMS); directional errors: horizontal (x), vertical (y), sagittal (z)], endpoint movement velocity and endpoint trajectory straightness. At the movement pattern level, measures were ranges of elbow extension, shoulder flexion and shoulder horizontal adduction as well as sagittal trunk displacement.

Endpoint tangential velocity was measured from the velocity vector, obtained by numerical differentiation of the x, y and z positions of the index finger marker. Movement beginning and end were defined as the times at which the velocity exceeded and remained above or fell and remained below 10% of the peak velocity respectively. RMS and directional (x, y, z) errors were computed between the endpoint position and the target center at movement end. Endpoint trajectory straightness was estimated using the index of curvature (IC) defined as the ratio of the length of the actual path traveled by the endpoint in 3D space to the length of an ideal straight line joining the initial and final endpoint positions [41]. An ideal straight line has an IC of 1, while a semicircle has an IC of 1.57.

Elbow extension was measured as the angle between the vectors formed by the wrist and elbow IREDs and elbow and ipsilateral shoulder IREDs (full elbow extension = 180°). Shoulder flexion was defined as the angle between the vector formed by the elbow and ipsilateral shoulder IREDs, and a straight line drawn through the ipsilateral shoulder vertical axis (arm alongside body = 0°). Shoulder horizontal adduction was measured as the angle between the vector formed by ipsilateral shoulder-elbow IREDs and the line traced by the contralateral-ipsilateral shoulder IREDs projected horizontally, where the zero position was defined as the fully abducted arm in line with the shoulders. Trunk displacement was measured as forward displacement (mm) of the IRED located on the sternum in the sagittal plane. Custom programs written in LabView^© ^(National Instruments Corp., Austin, TX) were used for kinematic analysis.

### Statistical analysis

Data normality was verified with Levene's tests. Mean values of arm kinematics while pointing when viewing the VE via the HMD and the SPS were compared using repeated measures ANOVAs with group (3 levels - control, stroke-mild, stroke-moderate-to-severe) being the fixed factor and viewing medium (2 levels - HMD, SPS) and targets (6 levels) being the repeated measures. Data were analyzed using SPSS^© ^(v17; SPSS Inc, Chicago, IL). Significance levels were set at α < 0.05. Since we were interested in the effects of the viewing media on movement performance, significant interactions involving the viewing media were primarily considered. For significant interactions, post-hoc testing using paired t-tests was carried out with Bonferroni corrections for multiple comparisons.

## Results

The mean age of the healthy (n = 10; 3 females) group was 53.6 ± 17.2 yrs and of the stroke group (n = 20; 3 females) was 66.2 ± 11.3 yrs. Demographic characteristics for the subgroups are listed separately in Table 1. All the subjects were comfortable wearing the HMD and none reported any side effects.

**Table 1:** Demographic and clinical characteristics of participants with stroke

Group	Subjects	Age (yrs)	Gender	Time since stroke (yrs)	Dominance	Side of hemiparesis	CM score (7)	FMA score (66)	CSI score (16)
	S1	74	M	6	Right	Left	4	51	4
	S2	70	M	6	Left	Right	5	51	8
	S3	62	M	5	Right	Left	5	52	7
Mild	S4	62	M	7	Right	Right	5	54	11
	S5	70	M	4	Right	Left	4	55	7
	S6	71	M	4	Right	Left	5	60	7
	S7	59	M	7	Right	Right	5	61	6
	S8	79	M	10	Right	Left	5	64	7
	S9	84	M	5	Right	Right	7	65	4
	S10	80	M	10	Right	Left	7	66	4

**Mean (SD)**		**71.1 (8.4)**		**6.4 (2.2)**			**5.2 (1.0)**	**57.9 (6.0)**	**6.5 (2.2)**

Group	Subjects	Age (yrs)	Gender	Time since stroke (yrs)	Dominance	Side of hemiparesis	CM score (7)	FMA score (66)	CSI score (16)

	S11	80	M	8	Right	Right	3	17	8
	S12	56	M	3	Left	Right	3	20	9
	S13	54	M	4	Left	Right	3	22	11
	S14	66	M	3	Right	Right	3	22	11
Moderate-to- -severe	S15	58	M	3	Right	Right	4	26	8
	S16	48	F	15	Right	Left	4	35	11
	S17	75	M	11	Right	Right	3	36	7
	S18	68	F	2	Right	Right	4	36	7
	S19	66	F	8	Right	Right	4	39	8
	S20	41	M	4	Right	Left	5	42	10

**Mean (SD)**		**61.2 (12.1)**		**6.1 (4.3)**			**3.6 (0.7)**	**29.5 (9.0)**	**8.5 (1.7)**

Abbreviations: F - Female; M - Male; CM - Arm section of Chedoke McMaster Stroke Assessment (7 = normal arm activity); CSI - Composite Spasticity Index (4 = Normal tone);FMA - Upper limb section of Fugl-Meyer Assessment (66 = full recovery)

### Motor performance variables

**Figure 2:**
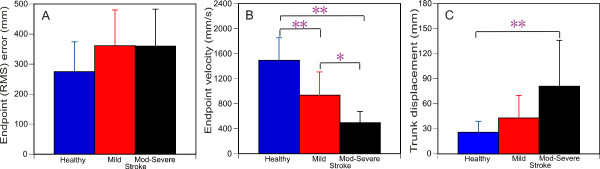
**Overall endpoint (root mean square, RMS) errors (A), endpoint velocity (B) and trunk displacement (C) for the three groups: healthy (blue), stroke-mild (red) and stroke-moderate-to-severe (black)**. Data are overall mean (SD) values across all 6 targets and both media. Asterisks indicate significance. * p < 0.01, ** p < 0.005

**Figure 3:**
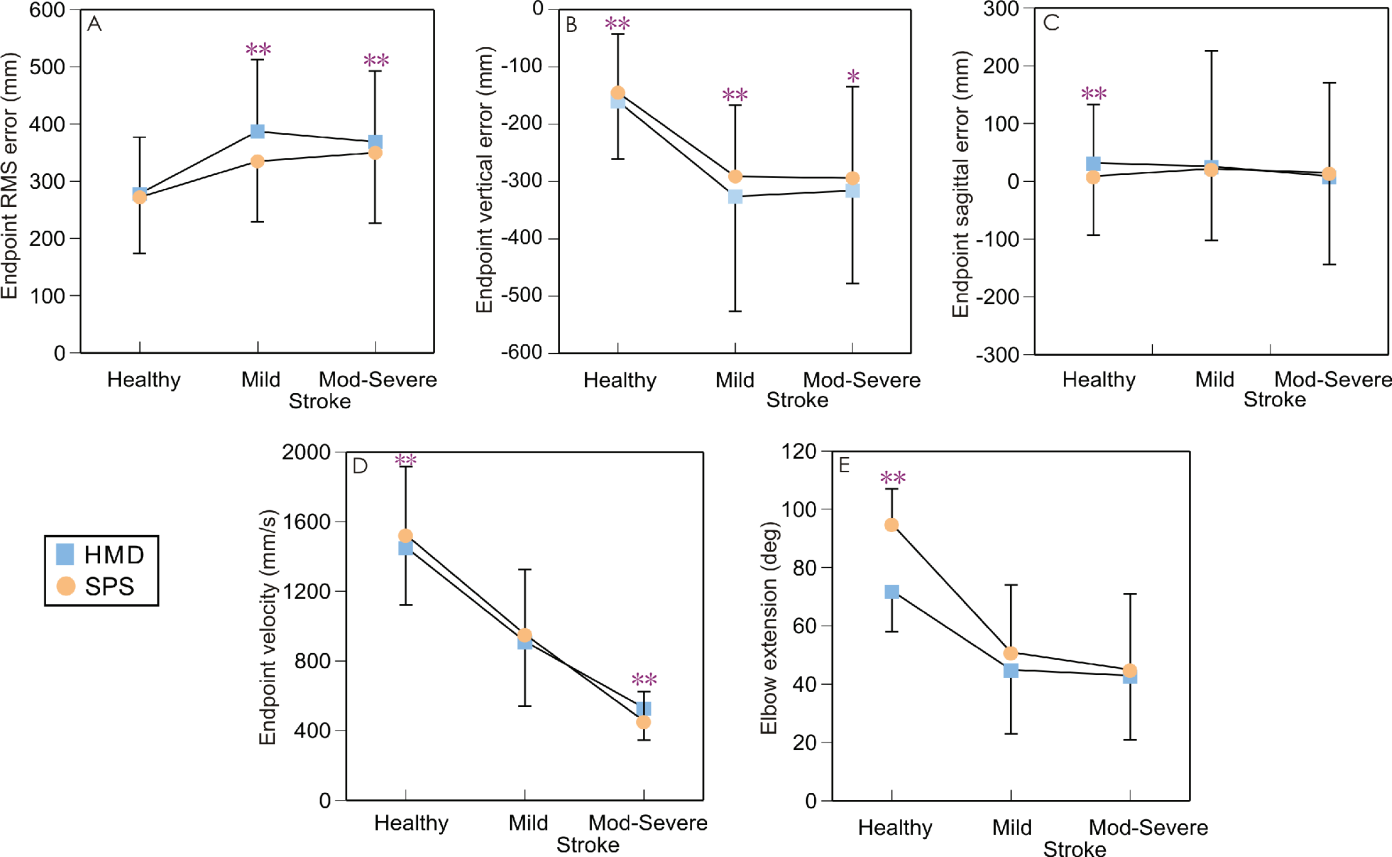
**Mean (SD) values across targets for total endpoint (root mean square, RMS) (A), vertical (B) and sagittal directional errors (C), endpoint velocities (D) and elbow extension ranges (E) for the three groups: healthy, stroke-mild and stroke-moderate-to-severe viewing the VE through the head mounted display (light blue squares) and screen projection system (orange circles)**. Asterisks indicate significance. * p < 0.01, ** p < 0.005

Stroke subjects tended to make less precise movements overall (one way ANOVA, p = 0.059) compared to the healthy subjects (Figure 2A). RMS errors were smaller with the SPS compared to the HMD (Figure 3A, group × viewing medium interaction; F_2,27 _= 6.539, p < 0.01). Post-hoc testing revealed the locus of this difference in the stroke-mild (t_59 _= 7.628, p < 0.005) and stroke-moderate-to-severe (t_59 _= 3.068, p < 0.005) subgroups.

**Figure 4:**
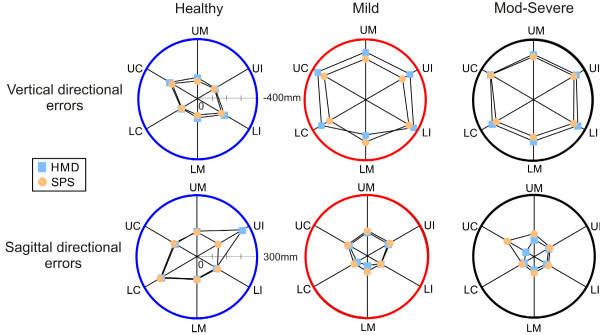
**Vertical (top row) and sagittal (bottom row) directional errors for the 6 targets for the three groups: healthy (blue circles), stroke-mild (red) and stroke-moderate-to-severe (black)**. Subjects viewed the VE through the head mounted display (light blue squares) and screen projection system (orange circles). Data are mean values in each group. Targets: UC - upper contralateral, UM - upper middle, UI - upper ipsilateral, LC - lower contralateral, LM - lower middle, LI - lower ipsilateral.

There were no differences between media across groups for errors in the horizontal direction. Overall group effects for vertical and sagittal directional errors are shown in Figure 3 (B, C) and their magnitude by target and group are shown in Figure 4. There were differences in the vertical (group × viewing medium × target interaction; F_10,48 _= 3.465, p < 0.05) and sagittal (group × viewing medium × target interaction; F_10,48 _= 4.542, p < 0.01) directions. In the vertical direction, all groups pointed below the target (Figure 3B) and errors were smaller for SPS compared to HMD across all targets: healthy (t_59 _= -4.259, p < 0.001), stroke-mild (t_59 _= -2.708, p < 0.001) and stroke-moderate-to-severe (t_59 _= -2.602, p < 0.016). In addition, vertical errors were greater for both targets located in the contralateral arm workspace and for the upper ipsilateral target (target main effect F_5,54 _= 57.41, p < 0.001) and this effect was most obvious in the mild stroke sub-group (Figure 4 top row).

In the sagittal direction, only healthy subjects had greater errors when viewing the scene with the HMD (32 mm) compared to SPS (9 mm; t_59 _= 3.258, p < 0.002; Figure 3C) and error primarily occurred for the upper ipsilateral target (Figure 4 bottom row, target main effect F_5,54 _= 264.29, p < 0.001).

The stroke subgroups made slower movements overall compared to the healthy subjects (F_2,27 _= 30.57, p < 0.001; Figure 2B). Movements were faster in the mild compared to the moderate-to-severe stroke subgroup (p < 0.01). The viewing media affected endpoint velocity (group × viewing medium; F_2,27 _= 3.64, p < 0.05) differently in each group. While the healthy group made faster movements when viewing the scene with the SPS (t_59 _= -3.701, p < 0.001), the stroke moderate-to-severe subgroup made faster movements when viewing the scene with the HMD (t_59 _= 5.884, p < 0.001; Figure 3D). Movement velocity in the mild stroke subgroup was not affected by the viewing medium.

Endpoint trajectory straightness values ranged from 1.08 - 1.36 for the healthy group, from 1.46 - 2.50 for the stroke-mild subgroup and from 1.62 - 2.42 for the stroke-moderate-to-severe subgroup. For all groups, endpoint trajectory straightness was not affected by either viewing medium or target location.

### Movement pattern variables

Overall, the moderate-to-severe stroke subgroup used more trunk displacement than the healthy group (F_2,27 _= 6.975, p < 0.005, Figure 2C). Elbow extension range of motion differed for movements made in different viewing media (target × viewing medium interaction; F_5,23 _= 3.431, p < 0.05) only for the lower middle target. Between media differences were found only for the healthy group who used more elbow extension when viewing with the SPS compared to the HMD (t_9 _= -4.701, p < 0.001; Figure 3E). For the other targets and movement pattern outcomes (shoulder flexion, shoulder horizontal adduction and trunk displacement), no significant interactions were found for target, group and/or viewing medium.

## Discussion

The effect of viewing a 3D VE through different media on performance of upper limb pointing movements was addressed in this study. We found some differences in motor performance and movement pattern kinematics depending upon the viewing medium used and the target location. Movements were less precise for the stroke subgroups when viewing the VE with the HMD compared to the SPS. We also found that there were larger vertical directional errors for all groups and sagittal directional errors for the healthy group when wearing the HMD. This suggests that subjects underestimated target height and distance when the environment was viewed via the HMD. Our results are in agreement with earlier studies evaluating the effects of reduced FOV on upper limb movements [23,42]. The HMD used in our study had a FOV of 30° vertical which is only 25% of normal [19]. Thus, reduction in vertical FOV may have affected the accuracy of the pointing movements.

In a previous study involving healthy and stroke subjects making pointing movements in a HMD-viewed VE and an equivalent PE, differences in precision occurred only for movements to contralateral targets with no significant group by environmental interaction [14]. In that study, subjects received visual (game score) and auditory feedback (knowledge of results and performance). Provision of feedback has been shown to improve egocentric distance perception in VEs viewed via a HMD despite limitations in vertical FOV to 35° [43] and reduce errors associated with pointing movements [44]. However, no feedback was provided in our study. Nevertheless, subjects may have received some feedback about target location because of the physical limit of the SPS. This may have assisted them in judging object affordances [45] and target distances better than with the HMD which may partly explain why movements were more precise with the SPS.

We also found differences between media in terms of movement velocity in healthy subjects and in stroke participants with moderate-to-severe upper limb impairment. The healthy group made slower movements when the VE was viewed through the HMD. This may be a result of limitations in FOV. Indeed, previous studies [14,21] have indicated that pointing movements were slower under conditions of reduced FOV. Another possibility is that subjects may have been less sure of where the target was located due to altered perception, but this was not directly measured in our study. On the other hand, the moderate-to-severe stroke group made faster pointing movements using the HMD. This result has not been reported previously. One explanation is that the use of excessive trunk displacement (Figure 2C) in this sub-group may have led to an increase in endpoint velocity [39,46]. However, there were no differences between the magnitudes of trunk displacement in either stroke group between viewing media and there was no correlation between trunk movement and endpoint velocity. Therefore, this observation requires further investigation.

No differences were noted between viewing media in terms of shoulder flexion, shoulder horizontal adduction or trunk displacement ranges. The only difference in movement performance variables was that healthy participants used less elbow extension for pointing towards the lower middle target using the HMD (72° ± 14°) compared to the SPS (95° ± 12°), a decrease of about 32%. Our results agree with those of Sahm and colleagues [23] indicating a 30% distance under-estimation for upper limb tasks performed by healthy subjects while viewing VEs using a HMD. The decreased range of elbow extension may be related to the perception of the target as being closer when viewing the scene through the HMD [47].

The weight of the HMD (1 kg) is one factor that may give rise to impaired distance estimation [25]. The weight on the head causes a modification of head and neck posture, which may increase the angular declination between eye level and the target causing distance underestimation [27]. The cap was lighter than the HMD. It remains to be estimated if pointing movement performance is influenced by wearing a heavier cap. This will help clarify whether the weight on the head is a confounding factor that impacts distance perception in addition to limitations in FOV when upper limb movements are performed. Whether viewing the VE using newer generation HMDs which are lighter in weight and have a wider FOV results in similar or different upper limb motor performance and movement pattern outcomes remains to be determined.

## Study Limitations

The goal of the study was only to compare the movement kinematics made under the two viewing conditions. Consequently, there are some limitations to the study. For example, the levels of comfort while wearing the HMD was not assessed, nor were neck kinematics. In addition, we did not assess the sense of presence and feeling of immersion of participants in the two environments which may have been interesting, since the use of HMDs may affect these factors [48] and impact on participant compliance and motivation to exercise. These factors should be addressed in future studies to help further elucidate the effect of wearing HMDs in VR environments for rehabilitation.

## Conclusions

We examined the effects of viewing a 3D VE through a HMD and SPS on upper limb pointing movements. In healthy subjects, movements were slower and less precise (vertical and sagittal directions) and subjects used less elbow extension when the VE was viewed with the HMD. In the stroke subjects, movements were less precise and there was a greater vertical directional error using the HMD. In addition, stroke subjects with moderate-to-severe hemiparesis made faster movements using the HMD. Wearing HMDs may result in neck-discomfort and may affect distance estimation. Thus, the use of SPS is suggested as a more comfortable and effective option to view VEs for rehabilitation post-stroke. Whether training in 3D fully immersive VE viewed via SPS results in similar or better outcomes as compared to conventional training also needs to be estimated. This will have implications for the design and use of VR rehabilitation applications to enhance upper-limb recovery post-stroke.

## Competing interests

The authors declare that they have no competing interests.

## Authors' contributions

SKS and MFL conceived of and designed the study and wrote the manuscript; SKS carried out data collection and analysis; MFL provided institutional affiliation and funding. Both authors read and approved the final manuscript.
